# Family income trajectory during childhood is associated with adiposity in adolescence: a latent class growth analysis

**DOI:** 10.1186/1471-2458-12-611

**Published:** 2012-08-05

**Authors:** Darla E Kendzor, Margaret O Caughy, Margaret Tresch Owen

**Affiliations:** 1 The University of Texas Health Science Center, School of Public Health, 6011 Harry Hines Boulevard, Dallas, TX 75390-9128, USA; 2 UT Southwestern Harold C. Simmons Comprehensive Cancer Center, Population Science and Cancer Control Program, Dallas, TX, USA; 3 The University of Texas at Dallas, School of Behavioral and Brain Sciences, 800 W. Campbell Road, Richardson, TX 75080-3021, USA

**Keywords:** USA, Socioeconomic status, Poverty, BMI, Waist circumference, Skinfold thickness, Obesity, Childhood

## Abstract

**Background:**

Childhood socioeconomic disadvantage has been linked with obesity in cross-sectional research, although less is known about how changes in socioeconomic status influence the development of obesity. Researchers have hypothesized that upward socioeconomic mobility may attenuate the health effects of earlier socioeconomic disadvantage; while downward socioeconomic mobility might have a negative influence on health despite relative socioeconomic advantages at earlier stages. The purpose of the current study was to characterize trajectories of family income during childhood, and to evaluate the influence of these trajectories on adiposity at age 15.

**Methods:**

Data were collected as part of the Study of Early Child Care and Youth Development (SECCYD) between 1991 and 2007 at 10 sites across the United States. A latent class growth analysis (LCGA) was conducted to identify trajectories of family income from birth to 15 years of age. Analyses of covariance (ANCOVAs) were conducted to determine whether measures of adiposity differed by trajectory, while controlling for relevant covariates.

**Results:**

The LCGA supported a 5-class trajectory model, which included two stable, one downward, and two upward trajectories. ANCOVAs indicated that BMI percentile, waist circumference, and skinfold thicknesses at age 15 differed significantly by trajectory, such that those who experienced downward mobility or stable low income had greater adiposity relative to the more advantaged trajectories. Conversely, upwardly mobile children and those with consistently adequate incomes had similar and more positive outcomes relative to the most disadvantaged trajectories.

**Conclusions:**

Findings suggest that promoting upward socioeconomic mobility among disadvantaged families may have a positive impact on obesity-related outcomes in adolescence.

## Background

The prevalence of childhood overweight and obesity remains alarmingly high, with recent estimates of over a third of children and adolescents measuring at ≥85^th^ percentile of body mass index [BMI; [1]. Unfortunately, obese children are more likely to suffer from a variety of medical problems including diabetes mellitus, features of the metabolic syndrome, polycystic ovarian syndrome, liver disease, sleep disturbances, and orthopedic disorders [for a review, see [2]. Obesity in childhood is likely to persist into adulthood [3,4] and overweight/obesity is one of the leading causes of death among adults [5]. Mortality from all causes is greater among overweight and obese adults than among those in the normal range of BMI [6].

Childhood socioeconomic disadvantage has been linked with obesity in cross-sectional research, although the relationship is complex and often varies by race/ethnicity [7-10]. Low socioeconomic status (SES) in childhood is also associated with adult obesity in longitudinal studies [for reviews, see [11,12], and initial research has indicated that childhood SES may have an influence on obesity beginning as early as adolescence [13]. Current conceptual models suggest that childhood SES may influence health later in life through a variety of physical and psychosocial exposures, which in turn influence psychological variables, health behavior, and physiology [for a review, see [14].

At least three broad theories, the timing or critical/sensitive period, accumulation/cumulative risk, and change/social mobility models, describe how exposure to low SES in childhood might influence health later in life [for reviews, see [14-16]. In the timing or critical/sensitive period model, SES-related factors are hypothesized to have the greatest impact on health during specific developmental stages (e.g., in utero, early childhood, adolescence). For example, Ziol-Guest et al. [17] reported that annual family income during the prenatal period and into the first year of life was associated with adult BMI, whereas income in later periods of childhood was not. In the accumulation/cumulative risk model, the intensity and duration of exposure to socioeconomic disadvantage are hypothesized to have the greatest influence on health. For example, Wells et al. [13] showed that children who spent a greater proportion of early childhood living in poverty experienced accelerated growth trajectories in adolescence based on age- and sex-adjusted BMI percentiles. However, less attention has been paid to the change/social mobility model in which upward socioeconomic mobility is hypothesized to attenuate the negative effects of earlier socioeconomic disadvantage, while downward socioeconomic mobility is hypothesized to have a negative influence on health despite relative socioeconomic advantages at earlier stages.

The purpose of the current study was to characterize common trajectories of family income during childhood and to evaluate the influence of these trajectories on adiposity in adolescence. It was hypothesized that children who experienced low income throughout childhood would have greater BMI percentile, waist circumference, and skinfold thickness at age 15 than those who experienced higher income throughout childhood. Children who experienced upward economic mobility were expected to have outcomes consistent with a reduced likelihood of obesity, while those who experienced downward economic mobility were expected to have outcomes consistent with greater obesity.

## Methods

The Study of Early Child Care and Youth Development (SECCYD) was a longitudinal study designed to examine the influence of child care experiences on social, emotional, intellectual, and language development; as well as physical growth and health of children [for more information, see [18,19]. Participants were enrolled at 10 sites across the U.S., and the study was completed in four phases starting at the birth of the child and continuing through 15 years of age. A total of 1,364 families were enrolled in Phase I in 1991, which continued from birth to 3 years of age and included assessments at 1, 6, 15, 24, and 36 months of age. During Phase II, 1,226 families were retained in the study. Phase II continued from 54 months through 1st grade, and included assessments at 54 months, kindergarten, and 1st grade. During Phase III, 1,061 families remained in the study. Phase III continued from 2nd through 6th grade, and included assessments at 2nd, 3rd, 4th, 5th, and 6th grades. By Phase IV, 1009 families remained in the study. Phase IV continued from 7th through 9th grade, and included assessments at 7th and 8th grades and at age 15 years. Data were collected by research assistants via home visits, visits to the child care facility, in a laboratory playroom, telephone calls, and mailed questionnaires. All study procedures were approved at the institutional reviews boards of each site (Temple University; University of Arkansas at Little Rock; Harvard University and Wellesley College; University of Califoria, Irvine; University of Kansas; University of North Carolina, Chapel Hill; University of Pittsburgh; University of Virginia; University of Washington, Seattle; University of Wisconsin, Madison), and informed consent was obtained from all participants. Information about inclusion/exclusion criteria, recruitment, enrollment, and the study protocol are presented in detail elsewhere [18,19]. Data collection ended in 2007, and study analyses presented in the current manuscript were completed in 2010 and 2011.

### Participants

Participants were mothers recruited from hospitals at 10 data collection sites who had just given birth and who 1) were at least 18 years of age, 2) were conversant in English, 3) planned to remain in the catchment area for at least 3 years, and 4) had a child without obvious disabilities who remained in the hospital for no more than 7 days following the birth.

### Measures

#### Race/ethnicity

Due to the limited number of individuals who endorsed non-white race/ethnicity, this variable was dichotomized into two categories: White/Caucasian and non-white.

#### Demographics/socioeconomic status

Maternal age and years of education were measured when the infant was 1 month of age. Income-to-needs ratio was calculated by dividing the self-reported total family income by the federal poverty threshold given the size of the family [e.g., the federal poverty threshold for a family of four in 1991 was $13,924; see [20]. Thus, an income-to-needs ratio of 1.0 indicates that the family was living at the poverty threshold. Given the low number of individuals living at or below the poverty threshold in the current sample, the income-to-needs ratio was dichotomized into low income (income-to needs ratio ≤ 2) or adequate income (income-to needs ratio > 2). This cut-point for low-income (i.e., 200% of the poverty threshold) was chosen because it has been used in previous research with the SECCYD data set [e.g., [21,22] and often serves as a criterion for qualifying for government aid [e.g., Child Health Insurance Program (CHIP)]. Income was measured at 13 assessment points beginning at 1 month of age and continuing through 15 years of age [i.e., [1,6,15,24], and 36 months (phase I); 54 months, kindergarten, and grade 1 (phase II); grades 3-6 (phase III); and 15 years of age (phase IV)]. All demographic and socioeconomic status data were collected via interview (through the 54 month assessment) or questionnaire (from kindergarten through age 15 assessments) either in the participant’s home or in the laboratory.

#### Anthropometric measures

Birth weight was measured in grams (g) at birth, and infants who weighed <2500 g were considered to be of low birth weight. BMI (kg/m²) was calculated based on height and weight measurements in the laboratory at age 15. BMI percentile was calculated based on normative data for gender and age [see [24] in order to facilitate the interpretation of BMI. Specifically, the healthy range of BMI percentile is considered to be 5-84%, the overweight range is 85-94%, and the obese range is 95% or greater based on gender and age [for more information about the use of BMI in chidren, see [24,25,26]. It is important to note that although BMI percentile is the most widely used measure of adiposity in children and is correlated with percent body fat, it is not a specific measure of body fat and is therefore also correlated with muscle and lean mass. Waist circumference and skinfold thickness measurements are also valid indicators of body fat [for a review, see [26] and were used here to supplement the information provided by BMI. Waist circumference (age 15) was measured in the laboratory in centimeters (cm). Subscapular and triceps skinfold thicknesses (age 15) were measured in the laboratory in millimeters (mm), and the values were summed to compute total skinfold thickness.

### Analysis plan

#### Latent class growth analysis

Mplus software version 5.21 was utilized to conduct a Latent Class Growth Analysis (LCGA). LCGA is a person-centered approach that aims to classify individuals into groups based on individual response patterns [see [27]. The purpose of the LCGA in this study was to characterize the optimal number of trajectories of family income over time (i.e., 1 month to 15 years of age). Models with 1-7 classes were estimated, beginning with the simplest model. Model fit for each of the 7 models was evaluated, in part, using the Bayesian Information Criterion (BIC), the Lo, Mendel, Rubin (LMR) statistic, and entropy values [for more information about model fit in LCGA, see [27]. Successive comparisons of the BIC were made beginning with the 1-class model, with lower values suggesting better model fit. The emergence of a non-significant LMR statistic suggested that the preceding model with one fewer class was to be preferred. Although there is no standard threshold by which to evaluate entropy, values near 1.0 are desirable. Finally, the models were visually inspected for their theoretical and practical coherence with a preference for simpler and more parsimonious models.

#### Analysis of covariance (ANCOVA)

A series of ANCOVAs were conducted to determine whether BMI, waist circumference, and skinfold thickness differed by income trajectory. Income trajectory, race/ethnicity, and gender were included as independent variables in the analyses, and birth weight, mother’s age, mother’s education, and income-to-needs-ratios at 1 month and 15 years of age were included as covariates. Interactions of gender and race/ethnicity with income trajectory were additionally evaluated after the main effects were examined. Individuals with missing covariates and/or outcome data at age 15 were excluded from the analyses.

## Results

### Participant characteristics

Participant characteristics (mother and child) for those included in the analyses are presented in Table 1.

**Table 1:** Participant characteristics

	***N***	**Mean (SD)**	**%**
Birth – 1 month of Age			
Birthweight (g)	1356	3489.6 (506.2)	-
Low Birth Weight (% < 2500 g)	1356	-	2.5
Female (%)	1356	-	48.5
White/Caucasian (%)	1356	-	76.3
Mother’s Age (Years)	1356	28.1 (5.6)	-
Mother’s Education (Years Completed)	1355	14.2 (2.5)	-
Income-to-Needs Ratio	1274	2.8 (2.7)	-
≤ Twice Poverty Threshold (%)	1274	-	45.8
15 Years of Age			
BMI Percentile	843	65.7 (26.7)	-
Overweight/Obese (% ≥ 85^th^ percentile)	843	-	31.0
Waist Circumference (cm)	829	75.6 (12.3)	-
Skinfold Thickness (mm)	866	27.7 (12.7)	-
Income-to-Needs Ratio	924	5.3 (5.8)	-
≤ Twice Poverty Threshold (%)	924	-	21.5

### Income trajectories

The LCGA included a total of 1356 participants, as 8 participants did not provide income data at any of the13 possible measurement points. BIC values declined substantially with the addition of each additional class (see Table 2). LMR values became non-significant for the 4-class model suggesting that the 3-class model may offer a good fit for the data (see Table 2). However, upon subsequent visual inspection it became apparent that the 3-class model did not include many of the expected and theoretically important trajectories. For example, individuals who experienced downward income trajectories were not represented. Therefore, the 5, 6, and 7 class models were additionally examined.

**Table 2:** **Indicators of model fit in the Latent Class Growth Analysis **^**a **^***(N*** **= 1356)**

**Classes**	**BIC**^**b**^	**Entropy**^**c**^	**LMR**^**d**^
1	17090.729	N/A	N/A
2	11378.546	0.91	*p* < .0001
3	10602.813	0.845	*p* < .0001
4	10383.154	0.83	*p* = .2257
**5**^**e**^	**10230.973**	**0.781**	***p*** **= .0001**
6	10191.308	0.76	*p* = .0037
7	10178.467	0.761	*p* = .0577

^**a**^**Latent class growth analysis is a person-centered approach that aims to classify individuals into groups based on individual response patterns.**

^**b**^**BIC = Bayesian Information Criterion; lower values suggest better model fit**

^**c**^**Entropy values close to 1 are desirable.**

^**d**^**LMR = Lo, Mendel, Rubin statistic; the emergence of a non-significant LMR suggests that the preceding model with one fewer class may be preferred.**

^**e**^**The 5-class model was chosen as the final version of the trajectory model, and the corresponding classes were utilized in all subsequent analyses.**

**Figure 1:**
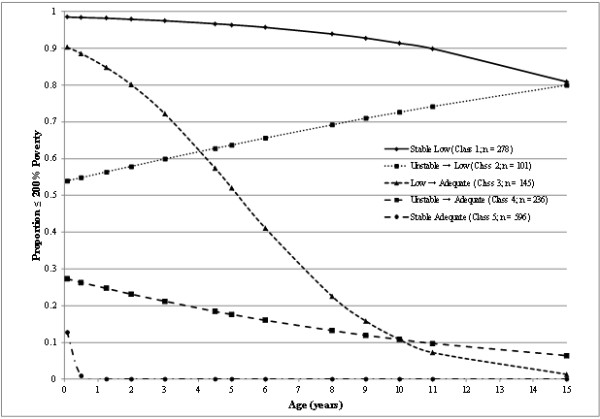
**Trajectories of household income from 1 month to 15 years of age.** Note: Assessment points are denoted in years (rather than grades or months) in order to more clearly depict the unequal spacing between assessments. Thus, the kindergarten assessment is shown at 5 years, the grade 1 assessment is shown at 6 years, the grade 3 assessment is shown at 8 years, the grade 4 assessment is shown at 9 years, the grade 5 assessment is shown at 10 years, and the grade 6 assessment is shown at 11 years.

The 5-class model (see Figure 1) included the following classes: 1) those who were likely to remain in the low income group over time (stable low income; *n* = 278), 2) those who initially experienced an unstable income status, but were likely to end up in the low income group (unstable → low income; *n* = 101), 3) those who began in the low income group, but were likely to end up in the adequate income group (low → adequate income; *n* = 145), 4) those who initially had a somewhat unstable income status, but who were likely to end up in the adequate income group (unstable → adequate income; *n* = 236), and 5) those who were likely to remain in the adequate income group over time (stable adequate income; *n* = 596). The 6-class model was very similar to the 5-class model, although included an additional class of individuals who initially had a low likelihood of being in the low income group, but for whom the likelihood increased slightly over time. The LMR value became non-significant for the 7-class model, which was therefore eliminated from consideration. The 5-class model was chosen as the final model primarily for simplicity and because the additional class in the 6-class model did not seem to be a useful or clearly delineated category, with 17.2% categorized as low-income at 1 month and increasing to 35.1% at 15 years. Participants differed significantly by income trajectory (based on the 5-class model) on all demographic, socioeconomic, and anthropometric characteristics with the exception of gender and proportion of low birth weight children (see Table 3).

**Table 3:** Participant characteristics by income trajectory

	**Stable Low (Class 1)**	**Unstable → Low (Class 2)**	**Low → Adequate (Class 3)**	**Unstable → Adequate (Class 4)**	**Stable Adequate (Class 5)**	***p***
Birth – 1 Month of Age						
Birth Weight (g)	3345 (469)	3481 (557)	3550 (526)	3550 (506)	3520 (498)	<.001
Low Birth Weight (% < 2500 g)	4.3	4.0	1.4	2.1	1.9	.161
Female Gender (%)	50.7	45.5	39.3	48.7	50.0	.175
White/Caucasian (%)	48.6	68.3	73.1	84.8	88.1	<.001
Mother’s Age (years)	23.6 (5.1)	26.3 (5.7)	25.1 (5.2)	29.1 (5.2)	30.9 (4.1)	<.001
Mother’s Education (years)	12.1 (1.8)	13.1 (1.9)	13.1 (2.2)	14.2 (2.1)	15.7 (2.1)	<.001
Income-to-Needs Ratio	0.5 (.6)	1.6 (1.1)	1.2 (1.0)	2.6 (2.1)	4.3 (2.8)	<.001
15 Years of Age						
BMI Percentile	76.2 (22.5)	75.2 (24.2)	66.5 (25.5)	64.3 (26.8)	60.6 (27.4)	<.001
Overweight/Obese (% ≥ 85^th^ percentile)	48.1	46.3	33.3	28.3	22.4	<.001
Waist Circumference (cm)	79.2 (15.0)	79.5 (12.6)	77.1 (12.1)	74.8 (11.0)	73.6 (11.3)	<.001
Skinfold Thickness (mm)	32.0 (15.7)	31.1 (13.5)	27.6 (13.2)	28.1 (12.2)	25.3 (10.9)	<.001
Income-to-Needs Ratio	1.4 (.9)	1.7 (1.1)	4.0 (3.4)	4.1 (3.2)	7.9 (7.0)	<.001

***Note:*Values in the table represent unadjusted means and standard deviations unless otherwise specified. Analyses of variance or chi-square analyses (for categorical outcomes) were used to evaluate differences between income trajectories.**

### Income trajectory and body mass index percentile

**Figure 2:**
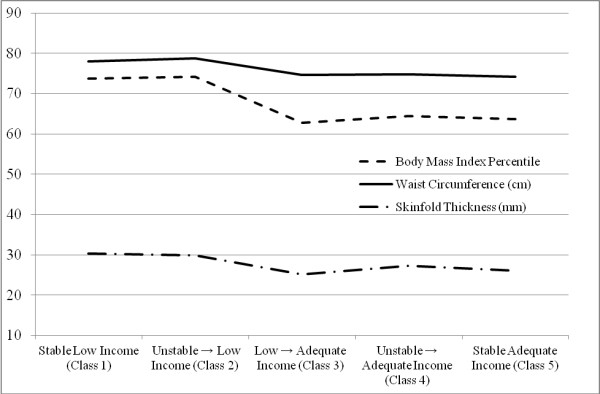
The influence of childhood income trajectory on body mass index percentile (N = 742), waist circumference (N = 732), and skinfold thickness (N = 766) at age 15 (adjusted values).

BMI percentile at age 15 differed significantly by income trajectory after controlling for relevant covariates F(4,730) = 3.610, *p* = .006, *N* = 742 (see Table 4). Using the least significant difference (LSD) test, post-hoc pairwise comparisons of income trajectories indicated that individuals in classes 1 (stable low income) and 2 (unstable → low income) had significantly greater BMI percentile than individuals in classes 3 (low → adequate income), 4 (unstable → adequate income), and 5 (stable adequate income; all *p*’s ≤ .014; see Figure 2). No significant differences in BMI percentile were found by gender or race/ethnicity. When interaction terms were added to the model, income trajectory did not significantly interact with gender or race/ethnicity.

**Table 4:** Analysis of covariance (ANCOVA) model of BMI percentile at age 15 years

**Source**	**Sum of Squares**	**df**	**Mean Square**	**F**	***p***	**Partial Eta²**
Mother’s Education (years)	1294.693	1	1294.639	1.955	.163	.003
Mother’s Age (years)	879.885	1	879.885	1.382	.249	.002
Birth Weight (g)	9556.052	1	9556.052	14.427	<.001	.019
Income-to-Needs Ratio at 1 month	175.500	1	175.500	.265	.607	.000
Income-to-Needs Ratio at 15 years	2910.011	1	2910.011	4.393	.036	.006
Gender	141.133	1	141.133	.213	.645	.000
Race/Ethnicity (white or non-white)	794.907	1	794.907	1.200	.274	.002
Income Trajectory	9564.026	4	2391.007	3.610	.006	.019
Error	483539.244	730	662.383			
Total	3689353.213	742				

### Income trajectory and waist circumference

Waist circumference at age 15 differed significantly by income trajectory after controlling for relevant covariates F(4,720) = 2.820, *p* = .024, *N* = 732 (see Table 5). Post-hoc pairwise comparisons of income trajectories using the LSD test indicated that individuals in classes 1 (stable low income) and 2 (unstable → low income) had significantly greater waist circumference than individuals in classes 4 (unstable → adequate income) and 5 (stable adequate income; all *p*’s ≤ .046; see Figure 2). In addition, individuals in class 2 (unstable → low income) had significantly greater waist circumference than those in class 3 (low → adequate income; *p* = .029). Waist circumference differed significantly by gender (*p* < .001), such that males had significantly greater waist circumference than females (78.185 cm vs. 73.961 cm). No differences in waist circumference were found by race/ethnicity. When interaction terms were added to the model, income trajectory did not significantly interact with gender or race/ethnicity.

**Table 5:** Analysis of covariance (ANCOVA) model of waist circumference at age 15 years

**Source**	**Sum of Squares**	**df**	**Mean Square**	**F**	***p***	**Partial Eta²**
Mother’s Education (years)	244.976	1	244.976	2.055	.152	.003
Mother’s Age (years)	141.883	1	141.883	1.190	.276	.002
Birth Weight (g)	2331.109	1	2331.109	19.553	<.001	.026
Income-to-Needs Ratio at 1 month	3.136	1	3.136	.026	.871	.000
Income-to-Needs Ratio at 15 years	237.666	1	237.666	1.993	.158	.003
Gender	3166.525	1	3166.525	26.560	<.001	.036
Race/Ethnicity (white or non-white)	13.470	1	13.470	.113	.737	.000
Income Trajectory	1344.964	4	336.241	2.82	.024	.015
Error	85839.242	720	119.221			
Total	4240690.667	732				

### Income trajectory and skinfold thickness

Skinfold thickness at age 15 differed significantly by income trajectory after controlling for relevant covariates, F(4,754) = 2.942, *p* = .020, *N* = 766 (see Table 6). Post-hoc pairwise comparisons of income trajectories using the LSD test indicated that skinfold thickness values for class 1 (stable low income) were significantly greater than values for classes 3 (low → adequate income; *p* = .006) and 5 (stable adequate income; *p* = .017; see Figure 2). Skinfold thickness differed significantly by gender, *p* < .001, such that females had greater values than males (31.255 mm vs. 24.164 mm, *p* < .001). No significant differences in skinfold thicknesses were found by race/ethnicity. When interaction terms were added to the model, income trajectory did not significantly interact with gender or race/ethnicity.

**Table 6:** Analysis of covariance (ANCOVA) model of skinfold thickness at age 15 years

**Source**	**Sum of Squares**	**df**	**Mean Square**	**F**	***p***	**Partial Eta²**
Mother’s Education (years)	475.336	1	475.336	3.473	.063	.005
Mother’s Age (years)	142.487	1	142.487	1.041	.308	.001
Birth Weight (g)	1048.682	1	1048.682	7.663	.006	.010
Income-to-Needs Ratio at 1 month	58.770	1	58.770	.429	.512	.001
Income-to-Needs Ratio at 15 years	876.120	1	876.120	6.402	.012	.008
Gender	9376.553	1	9376.553	68.516	<.001	.083
Race/Ethnicity (white or non-white)	145.878	1	145.878	1.066	.302	.001
Income Trajectory	1610.684	4	402.671	2.942	.020	.015
Error	103186.566	754	136.852			
Total	698009.250	766				

### Missing data

Of the 1356 participants for whom income trajectories were calculated, 45.3% (*n* = 614) were excluded from the analyses in which BMI percentile was the outcome due to missing BMI percentile and/or covariates. Similarly, up to 46% of the sample was excluded from the analyses where waist circumference (624 missing) and skinfold thickness (590 missing) at age 15 were the outcomes. Participants with missing data in the analyses where BMI percentile was the outcome differed in several ways from those who did not have missing data. Participants with missing data had significantly lower income-to needs ratio at 1 month of age (2.55 vs. 2.91, *p* = .018), were more likely to be non-white (50.8% vs. 43.6%, *p* = .023); and to have mothers who were younger (27.19 vs. 28.90 years, *p* < .001) and less educated (13.85 vs. 14.55 years, *p* < .001). Significant differences in the proportion of missing data by income trajectory were found (*p* < .001), such that those in the stable low income trajectory (class 1) had the highest proportion of missing data (65.5% missing), followed by the low → adequate (class 3; 51.7% missing), unstable → adequate (class 4; 40.3% missing), stable adequate (class 5; 38.9% missing), and unstable → low (class 2; 29.7% missing) income trajectories. Missing data did not vary by gender or birth weight category (i.e., <2500 g), and there was no difference in average birth weight by missing status. Differences by missing status in the analyses of the other outcome variables (waist circumference and skinfold thickness) were similar to those found by missing status on BMI percentile (results available upon request).

## Discussion

The primary purpose of the current study was to characterize common trajectories of family income during childhood and to evaluate the influence of these trajectories on adolescent adiposity. Findings suggest five income trajectories, including two stable trajectories (stable low income and stable adequate income), one trajectory indicating downward mobility (unstable → low income), and two trajectories indicating upward mobility (low → adequate income; unstable → adequate income). Overall, results indicate that the stable low income (class 1) and unstable → low income (class 2) trajectories were associated with significantly greater BMI percentile, waist circumference, and skinfold thickness than the more advantage trajectories. Thus, findings contribute to our understanding of the change model, in that downwardly mobile children were found to have worse obesity-related outcomes than those who were upwardly mobile. Notably, upwardly mobile individuals had similar outcomes to those who followed the stable adequate income trajectory, and downwardly mobile individuals had similar outcomes to those who followed the stable low income trajectory.

Individuals who followed the stable low (class 1) and unstable → low (class 2) income trajectories differed in a variety of ways from those who followed the other more economically advantaged trajectories. Children who followed the stable low income (class 1) trajectory weighed less at birth, and were more likely to be non-white and to have an income-to-needs ratio below the low income threshold. Additionally, these children were more likely to have mothers who were younger and had completed fewer years of education. Children who followed the unstable → low income trajectory (class 2) had characteristics that were very similar to those of the children who followed the stable low income trajectory (class 1). Thus, families of children who followed more disadvantaged income trajectories may have been less able to improve their socioeconomic circumstances or to achieve upward mobility due to low maternal education, low family income at birth, and perhaps factors associated with non-white race/ethnicity (e.g., employment discrimination and other marginalization factors).

The current study has several strengths and limitations. In particular, the prospective design allowed for the evaluation of the influence of income trajectories over 15 years on adolescent adiposity at age 15. Income status was measured repeatedly across 13 occasions over the 15-year study, thereby providing a more accurate representation of childhood SES. Previous studies that have examined the influence of childhood SES on health outcomes in adulthood have been criticized for failure to control for childhood health status and adult SES concurrent with the health outcome [see [14]. Such concerns were addressed in the current study by including birth weight as a covariate in the analyses in order to account for body size at birth. This is important given that higher birth weight is associated with greater BMI and obesity in adulthood [28,29], with the caveat that children at the lowest end of the birth weight spectrum may also be more likely to experience increased abdominal adiposity and reduced lean body mass in later life [30]. Income-to-needs ratio at 1 month and 15 years of age were additionally included as covariates in the analyses to account for the influences of both initial and ending levels of SES. Unfortunately, the proportion of missing data for the outcome variables was high, although similar to the rates of missing data in other longitudinal studies e.g., [3,31-33]. Further, participants who were excluded from the analyses due to missing data had lower income-to-needs-ratio at 1 month of age, were more likely to be non-white and to follow the stable low income trajectory, and had mothers who were younger and less educated. Thus, children from the lowest SES backgrounds may be somewhat underrepresented in the current study. It is difficult to say with any certainty how differences in missing outcome data might impact the findings. However, it is plausible that differences in adiposity across the trajectories might have been even greater if more of the most economically disadvantaged children (e.g., class 1; stable low income) were included in the analyses.

The findings of the current study may have important policy implications. Increasing the availability of funding for education and job training would allow many economically disadvantaged parents to successfully compete for higher paying jobs, thereby promoting upward socioeconomic mobility and child health. Parent participation in educational and/or training sessions have a beneficial impact on obesity-related outcomes [34], and such approaches warrant consideration for use among economically disadvantaged families. It is of particular importance to provide parents with guidance about the how the health needs of their children might be met with limited financial resources (e.g., purchasing healthy foods on small budget). The diet and physical activity levels of economically disadvantaged children may be directly targeted through programs that are delivered in a school setting [35,36]. Increased taxation of high-sugar/calorie beverages that contribute to obesity might also be an effective approach, as higher differential soda tax rates in schools are associated with lower soda consumption among children from low income families [37].

Future studies are needed to identify the obesity-related physiological, environmental, psychological, and behavioral factors associated with both unremitting economic disadvantage and downward socioeconomic mobility in children. Researchers have described a variety of mechanisms through which SES may influence health across the lifespan, including differential access to health care, environmental exposures, health behavior, and differential exposure to stress [38]. Many of these factors may also play an important role in the relation between childhood SES and adolescent obesity. Increasing our understanding of the socioeconomic influences on health will ultimately contribute to the elimination of health disparities across the lifespan.

## Conclusions

Findings suggest that changes in socioeconomic status during childhood may influence adiposity in adolescence. Specifically, the trajectories that ended with a higher proportion of children in low-income families showed greater adiposity at age 15, while the trajectories ending with a lower proportion of children in low-income families showed less adiposity. BMI percentile, waist circumference, and skinfold thickness at age 15 differed significantly by trajectory, such that those who experienced downward mobility or stable low income had greater adiposity relative to the more advantaged trajectories. Conversely, upwardly mobile children and those with consistently adequate incomes had similar and more positive outcomes relative to the most disadvantaged trajectories. Overall, findings suggest that promoting upward socioeconomic mobility among disadvantaged families may have a positive impact on obesity-related outcomes in adolescence.

## Competing interests

The authors declare that they have no competing interests.

## Authors’ contributions

DEK was the primary author of the manuscript, contributed to the study conceptualization and design, conducted all statistical analyses, and contributed to interpretation of the data. MOC and MTO each contributed to the study conceptualization and design, interpretation of the data; and manuscript preparation, review, and revision. All authors read and approved the final manuscript.

## Pre-publication history

The pre-publication history for this paper can be accessed here:

http://www.biomedcentral.com/1471-2458/12/611/prepub
